# Importance of confirming the underlying diagnosis in patients with myocardial infarction and non-obstructive coronary arteries (MINOCA): a single-centre retrospective cohort study

**DOI:** 10.1186/s12872-021-02176-2

**Published:** 2021-07-28

**Authors:** T. F. S. Pustjens, A. Meerman, N. P. A. Vranken, A. W. Ruiters, B. Gho, M. Stein, M. Ilhan, L. Veenstra, P. Winkler, Á. Lux, S. Rasoul, A. W. J. van ‘t Hof

**Affiliations:** 1 Department of Cardiology, Zuyderland Medical Centre, P.O. Box 5500, 6130 MB Sittard-Geleen, Heerlen, The Netherlands; 2 Department of Cardiology, Maastricht University Medical Centre, Maastricht, The Netherlands; 3 Cardiovascular Research Institute Maastricht (CARIM), Maastricht University, Maastricht, The Netherlands

**Keywords:** MINOCA, Acute coronary syndrome, Outcome

## Abstract

**Background:**

Many patients with myocardial infarction with non-obstructive coronary arteries (MINOCA) are discharged without a known aetiology for their clinical presentation. This study sought to assess the effect of this ‘indeterminate MINOCA’ diagnosis on the prevalence of recurrent cardiovascular events and presentations to the Cardiac Emergency Department (CED).

**Methods:**

We retrospectively analysed all patients meeting the diagnostic MINOCA criteria presenting at a large secondary hospital between January 2017 and April 2019.

**Participants:**

Patients were divided into the (1) ‘indeterminate MINOCA’, or (2) ‘MINOCA with diagnosis’ group. The primary outcome was the occurrence of major adverse cardiac events (MACE) defined as the composite of all-cause mortality, non-fatal myocardial infarction, stroke and any revascularisation procedure. Secondary outcomes were all recurrent visits at the CED, and MACE including unplanned cardiac hospitalisation.

**Results:**

In 62/198 (31.3%) MINOCA patients, a conclusive diagnosis was found (myocardial infarction, (peri)myocarditis, cardiomyopathy, or miscellaneous). MINOCA patients with a confirmed diagnosis were younger compared to those with an indeterminate diagnosis (56.7 vs. 62.3 years, *p* = 0.007), had higher maximum troponin-T [238 ng/L vs. 69 ng/L, *p* < 0.001] and creatine kinase (CK) levels [212U/L vs. 152U/L, *p* = 0.007], and presented more frequently with electrocardiographic signs of ischaemia (71.0% vs. 47.1%, *p* = 0.002). Indeterminate MINOCA patients more often showed recurrent CED presentations (36.8% vs. 22.6%, *p* = 0.048), however the occurrence of cardiovascular events was equal (8.8 vs. 8.1%, *p* = 0.86). Multivariable analysis showed that elevated levels of troponin-T and CK, ST-segment deviation on electrocardiography, reduced left ventricular ejection fraction, regional wall motion abnormalities, and performance of additional examination methods were independent predictors for finding the underlying MINOCA cause.

**Conclusions:**

Only in one-third of MINOCA patients a conclusive diagnosis for the acute presentation was identified. Recurrent CED visits were more often observed in the indeterminate MINOCA group, while the occurrence of cardiovascular events was similar across groups.

***Trial registration*:**

Retrospectively registered

## Background

Myocardial infarction with non-obstructive coronary arteries (MINOCA) occurs frequently in patients presenting with acute coronary syndrome (ACS) and represents a conundrum of many possible underlying aetiologies [1, 2]. Previous research has showed that additional examination consisting of cardiac magnetic resonance imaging (CMR), intravascular imaging and provocative testing provide added value in identifying the underlying diagnosis [3–7]. However, up to 75% of MINOCA patients are discharged without a definitive diagnosis responsible for the clinical event [8, 9]. Consequently, this may lead to patients being inappropriately or insufficiently treated, while the patient is left with uncertainty regarding their health status and prognosis.

Expectedly, this illness uncertainty may lead to recurrent visits at the Cardiac Emergency Department (CED), in part due to ongoing or recurrent undefined symptoms with a potential negative impact on morbidity and quality-of-life. Little is known about outcome in MINOCA patients per specific underlying cause, since in current literature, presented outcome data mostly reflect a heterogeneous MINOCA group [10]. Since the term MINOCA should rather be considered as a working diagnosis which should trigger physicians to initiate further evaluation, it remains important to identify the underlying mechanism(s) in individual patients in order to enable initiation of appropriate treatments [6, 11]. Therefore, the current study primarily aimed to assess the effect of an indeterminate MINOCA working diagnosis, and secondly, a confirmed MINOCA event on the prevalence of cardiovascular events and recurrent CED visits.

## Methods

### Study population and design

In this single-centre retrospective cohort study, all consecutive patients undergoing invasive coronary angiography (ICA) between January 2017 and April 2019 were evaluated.

Patients meeting the diagnostic criteria for MINOCA were included, i.e., patients complying with (1) the acute myocardial infarction (AMI) criteria as defined by the ‘Fourth universal definition of myocardial infarction’; (2) non-obstructive coronary arteries (no coronary stenosis ≥ 50% in any potential infarct-related artery); and (3) no other clinically overt specific cause for the acute presentation [1, 12]. The degree of stenosis was estimated by the treating interventional cardiologist. In case of a moderate coronary lesion (stenosis < 50%), patients remained eligible only in presence of a negative fractional flow reserve assessment. Patients who underwent ICA for any reason other than suspicion of ACS or did not meet the MINOCA criteria, were excluded.

If the underlying mechanism of the MINOCA event was found, based on a thorough review by two investigators (AM and TP) of all available clinical records, the patient was allocated to the ‘MINOCA with diagnosis’ group. Remaining cases were allocated to the ‘indeterminate MINOCA’ group. Any disagreements were resolved by a third physician (SR).

Due to the retrospective nature of this study, no informed consent was deemed necessary by the medical ethical committee of the Zuyderland Medical centre (METCZ). This study was conducted according to the principles of the Declaration of Helsinki and had been approved by the METCZ.

### Data collection

Data on individual patient characteristics, cardiovascular risk factors, laboratory results, results from electrocardiography as well as invasive and non-invasive imaging procedures were derived from the electronic health record system.

### Outcome definition

The primary outcome of the current study was the occurrence of major adverse cardiac events (MACE) defined as the composite of all-cause mortality, non-fatal myocardial infarction, stroke, and any revascularisation procedure performed during follow-up with a minimum follow-up duration of one year. Secondary outcomes consisted of all recurrent CED visits, and unplanned cardiac hospitalisation.

Follow-up data and events were obtained in April 2020 from the electronic health record system and planned visits with the treating cardiologist.

### Statistical analysis

Descriptive statistics for continuous variables are presented as a mean with standard deviation (SD) or median and interquartile range [IQR], depending on data distribution. Either an unpaired Student T-test or Mann–Whitney U test was used to analyse differences in continuous parameters across study groups.

Categorical data are reported as frequency values and assessed using Pearson’s X^2^-test or the Fisher’s exact test where appropriate.

In order to identify the predictors independently associated with finding an underlying cause for the MINOCA event and recurrent CED visits, multivariable logistic regression analysis was performed to derive adjusted odds ratios (OR) with corresponding 95% confidence intervals. Predictors included in the logistic regression models were selected based on the results of univariable analysis, employing a *p* < 0.30 threshold for inclusion. Age and gender were regarded as clinically important confounders and included in the model irrespective of p-value. Survival analysis was carried out using Kaplan–Meier curves to analyse the time to a recurrent CED visit, in which group differences were analysed using the log-rank test.

A two-tailed *p* value < 0.05 was considered statistically significant for all tests. Statistical analysis was performed using SPSS version 26.0.

## Results

**Fig. 1:**
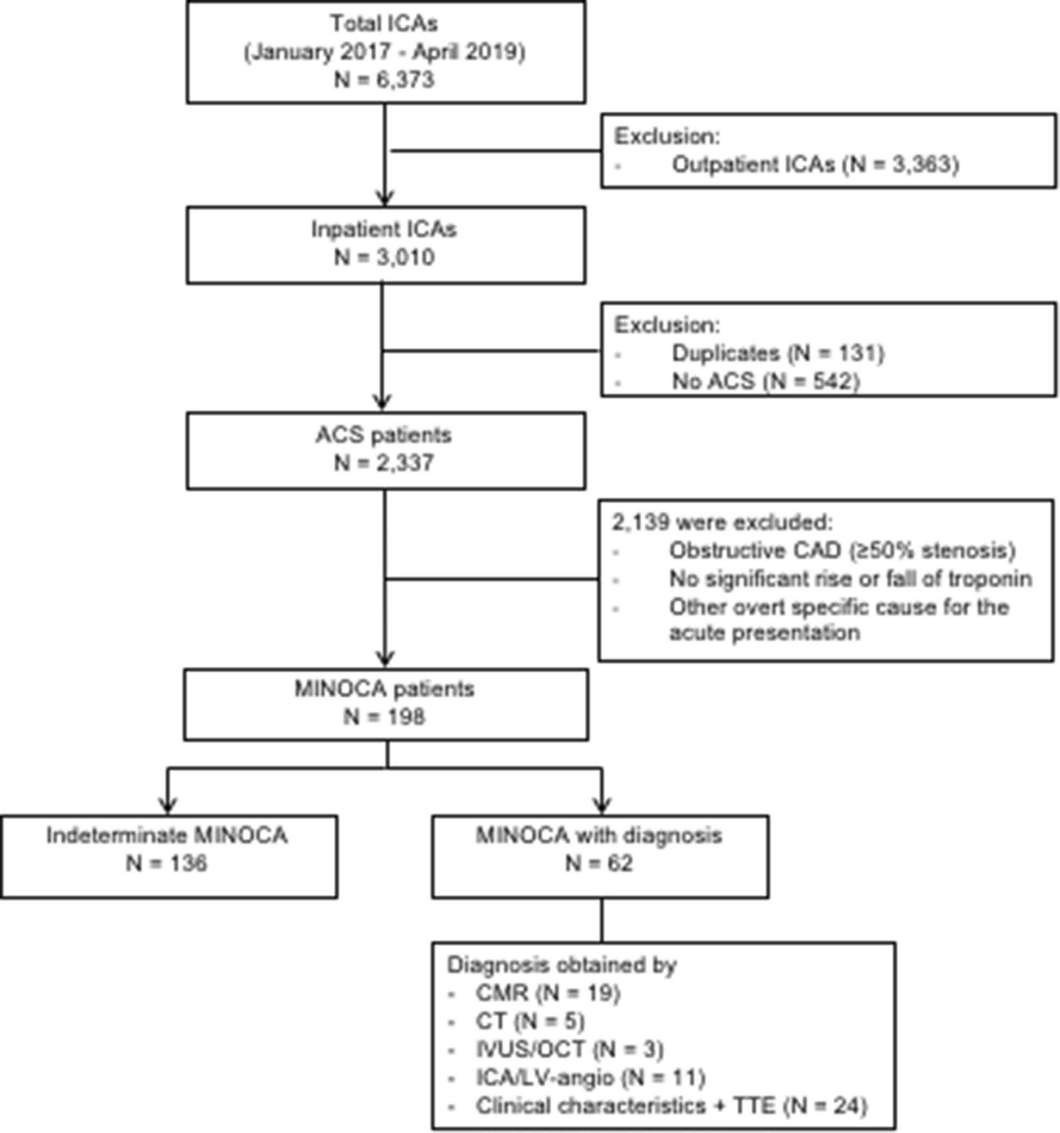
Flowchart of included MINOCA patients. ICA: invasive coronary angiography; ACS: acute coronary syndrome; CAD: coronary artery disease; MINOCA: myocardial infarction with non-obstructive coronary arteries; CMR: cardiac magnetic resonance imaging; CT: computed tomography; IVUS: intravascular ultrasound; OCT: optical coherence tomography; LV-angio: left ventricular angiogram; TTE: transthoracic echocardiogram

Between January 2017 and April 2019, 2,337 ICA procedures with ACS as primary indication were performed. A total of 198 (8.5%) individual patients fulfilled the MINOCA criteria (Fig. 1) [1, 2].

### Clinical characteristics

Patient characteristics are presented in Table 1. Of 198 MINOCA patients, 62 (31.3%) had MINOCA with a conclusive diagnosis, which in 38.7% (24/62) was found to be a confirmed AMI, cardiomyopathy in 27.4% (17/62), (peri)myocarditis in 22.6% (14/62) and another cause for the event (e.g., coronary spasm, spontaneous coronary artery dissection, pulmonary embolism, or aortic dissection) in 11.3% (7/62). The median time to a diagnosis was 2 days [0–37.5] which was similar to the indeterminate MINOCA group (2 days [1.0–3.0]; *p* = 0.068). In merely 10/198 cases, a diagnosis or indeterminate diagnosis was made > 60 days after the index hospitalisation. Compared to those with an indeterminate MINOCA diagnosis, patients with a conclusive diagnosis were younger (56.7 vs. 62.3 years, *p* = 0.007), less likely to have a history of previous AMI or percutaneous coronary intervention (PCI) (3.2% vs. 19.9%, *p* = 0.002; 1.6% vs. 11.8%, *p* = 0.018), and showed higher maximum serum levels of troponin-T [238 ng/L vs. 69 ng/L, *p* < 0.001] and creatine kinase (CK) [212U/L vs. 152U/L, *p* = 0.007]. Overall, electrocardiographic signs of myocardial ischaemia were more frequently observed in the MINOCA with diagnosis group, which was mainly driven by the presence of ST-elevations (43.5% vs. 17.6%, *p* < 0.001). The occurrence of ST-depression and negative T-wave changes did not differ between both groups (4.8% vs. 12.5%, *p* = 0.097 and 22.6% vs. 16.9%, *p* = 0.34 respectively). A left ventricular ejection fraction (LVEF) ≤ 45% was more prevalent in patients with a conclusive diagnosis compared to the indeterminate MINOCA group (36.0% vs. 8.4%, *p* < 0.001).

**Table 1:** Demographic characteristics in MINOCA patients

	Total	Indeterminate MINOCA	MINOCA with diagnosis	*p* value
	n = 198	n = 136	n = 62	
Mean age, years	60.5 ± 13.8	62.3 ± 12.9	56.7 ± 14.9	0.007
Female gender	112 (56.6)	80 (58.8)	32 (51.6)	0.34
BMI, kg/m^2^	26.8 [24.2–30.4]	26.8 [24.2–30.4]	26.8 [24.1–30.3]	0.93
*Cardiovascular risk factors*				
Current smoker	58 (29.3)	37 (27.2)	21 (33.9)	0.34
Diabetes Mellitus	37 (18.7)	27 (19.9)	10 (16.1)	0.53
Hypertension	84 (42.4)	64 (47.1)	20 (32.3)	0.051
Hypercholesterolemia	55 (27.8)	42 (30.9)	13 (21.0)	0.15
Previous AMI	29 (14.6)	27 (19.9)	2 (3.2)	0.002
Previous PCI	17 (8.6)	16 (11.8)	1 (1.6)	0.018
Previous stroke	14 (7.1)	11 (8.1)	3 (4.8)	0.56
Family history of CVD	75 (37.9)	52 (38.2)	23 (37.1)	0.88
Gout or rheumatic arthritis	16 (8.1)	14 (10.3)	2 (3.2)	0.091
COPD	22 (11.1)	16 (11.8)	6 (9.7)	0.67
Atrial fibrillation	14 (7.1)	11 (8.1)	3 (4.8)	0.56
Sleep apnea	8 (4.0)	6 (4.4)	2 (3.2)	1.00
Systolic blood pressure, mmHg	145.0 [126.5–165.0]	147.5 [125.0–171.5]	135.0 [128.8–151.8]	0.039
*Laboratory findings*				
Maximum hsTnT, ng/L	91.0 [43.8–332.5]	68.5 [34.5–219.8]	238.0 [66.3–655.0]	< 0.001
Maximum CK, U/L	168.0 [100.5–274.5]	152.0 [95.0–243.8]	212.0 [112.8–401.0]	0.007
Creatinine, µmol/L	80.4 ± 22.2	80.3 ± 22.4	80.6 ± 21.7	0.95
LDL, mmol/L	2.5 ± 0.9	2.6 ± 0.9	2.2 ± 0.9	0.009
ST-deviation at presentation	108 (54.5)	64 (47.1)	44 (71.0)	0.002
Absence of coronary atherosclerosis on ICA	67 (33.8)	38 (27.8)	29 (46.8)	0.009
Echocardiography performed	155 (78.3)	102 (75.0)	53 (85.5)	0.097
LVEF ≤ 45%	26 (17.9)	8 (8.4)	18 (36.0)	< 0.001
Wall motion disturbances	44 (29.3)	26 (51.0)	18 (18.2)	< 0.001
*Additional imaging performed*				
CMR	38 (19.2)	12 (8.8)	26 (41.9)	< 0.001
CT-angiography	25 (12.6)	11 (8.1)	14 (22.6)	0.004
Left ventricular angiography	24 (12.1)	12 (8.8)	12 (19.4)	0.035
*Medication at discharge*				
Acetylsalicylic acid	143 (72.2)	99 (72.8)	44 (71.0)	0.79
P2Y12 inhibitor	116 (58.6)	86 (63.2)	30 (48.4)	0.049
DAPT	106 (53.5)	77 (56.6)	29 (46.8)	0.20
Oral anticoagulation	22 (11.1)	14 (10.3)	8 (12.9)	0.59
Beta-blocker	131 (66.2)	94 (69.1)	37 (59.7)	0.19
ACE-inhibitor or ARB	109 (55.1)	77 (56.6)	32 (51.6)	0.51
Statin	138 (69.7)	99 (72.8)	39 (62.9)	0.16

Values are *n* (%), mean ± SD, or median [interquartile range]

MINOCA: myocardial infarction with non-obstructive coronary arteries; BMI: Body Mass Index; AMI: acute myocardial infarction; PCI: percutaneous coronary intervention; CVD: cardiovascular disease; COPD: chronic obstructive pulmonary disease; hsTnT: high-sensitivity troponin T; CK: creatine kinase; LDL: low-density lipoprotein; ICA: invasive coronary angiography; LVEF: left ventricular ejection fraction; CMR: cardiovascular magnetic resonance; CT-angiography: computed tomography angiography; DAPT: dual antiplatelet therapy; ACE: angiotensin converting enzyme; ARB: angiotensin II receptor blocker

CMR was performed in 19.2% of the MINOCA patients, with a median time between clinical presentation and CMR of 30 days [16.8–64.8]. Performance of CMR resulted in a conclusive diagnosis in 50% of the cases. Of them, 64% showed to have an AMI based on territorial subendocardial or transmural late gadolinium enhancement, in 20% cardiomyopathy based on specific tissue characterization was found, and the remaining 16% of patients were diagnosed with myocarditis based on the Lake Louise Criteria [13]. Computed tomography (CT) angiography was performed in 25 patients and revealed a conclusive diagnosis in 20% of the patients; 4 showed pulmonary embolism, and 1 aortic dissection.

In 24 MINOCA patients, left ventriculography was performed directly following ICA revealing Takotsubo syndrome in 25% of cases.

Following hospital discharge, patients with an indeterminate MINOCA were more likely to receive treatment with P2Y12-inhibitors (63.2% vs. 48.4%, *p* = 0.049), whereas the use of acetylsalicylic acid, oral anticoagulants, beta-blockers, angiotensin-converting enzyme (ACE) inhibitors or angiotensin-II receptor blockers (ARB), and statins were equally prescribed (Table 1).

### Clinical outcomes

During a median follow-up time of 719 [504–888] days, MACE occurrence was similar across MINOCA groups (8.8% vs. 8.1%, *p* = 0.86), with a median time to MACE occurrence of 213 [53–442] days (Table 2). None of the patients were lost-to-follow-up and no events occurred before a final diagnosis or indeterminate diagnosis was made. The total number of events during follow-up was: 6 deaths (3.0%), 9 new non-fatal MIs (4.5%), 2 coronary revascularization procedures (1.0%), and 4 ischemic strokes (2.0%). In the MINOCA with diagnosis group, 3 out of 5 MACE occurred in patients with confirmed AMI.

**Table 2:** Clinical outcome

	Total	Indeterminate	MINOCA	*p *value
	n = 198	MINOCA	with diagnosis	
		n = 136	n = 62	
Days follow-up	719 [504–888]	749 [504–889]	640 [501–896]	0.73
Days to diagnosis	2.0 [1.0–4.0]	2.0 [1.0–3.0]	2.0 [0–37.5]	0.068
MACE	17 (8.6)	12 (8.8)	5 (8.1)	0.86
All-cause mortality	6 (3.0)	4 (2.9)	2 (3.2)	1.00
Non-fatal myocardial infarction	9 (4.5)	8 (5.9)	1 (1.6)	0.28
Any revascularisation	2 (1.0)	2 (1.5)	0 (0.0)	1.00
Stroke	4 (2.0)	2 (1.5)	2 (3.2)	0.59
Recurrent visit at the CED	64 (32.3)	50 (36.8)	14 (22.6)	0.048
Cardiac aetiology for recurrent visit	52 (81.3)	42 (84.0)	10 (71.4)	0.44
Acute coronary syndrome	9 (14.1)	8 (16.0)	1 (7.1)	0.67
Stable angina pectoris	22 (34.4)	17 (34.0)	5 (35.7)	1.00
Other cardiac reason	21 (32.8)	17 (34.0)	4 (28.6)	1.00
Recurrent cardiac hospitalisation	24 (12.1)	19 (14.0)	5 (8.1)	0.24
Participation in cardiac rehabilitation programme	63 (31.8)	36 (26.5)	27 (43.5)	0.017

Values are *n* (%), or median [interquartile range]

MINOCA: myocardial infarction with non-obstructive coronary arteries; MACE: major adverse cardiac events; CED: Cardiac Emergency Department; non-STEMI: non-ST-elevation myocardial infarction

The two revascularization procedures both concerned new obstructive coronary artery disease of the left anterior descending artery 7 and 14 months after the index procedure respectively. Both patients already had diffuse nonobstructive coronary artery disease at the index coronary angiography.

**Fig. 2:**
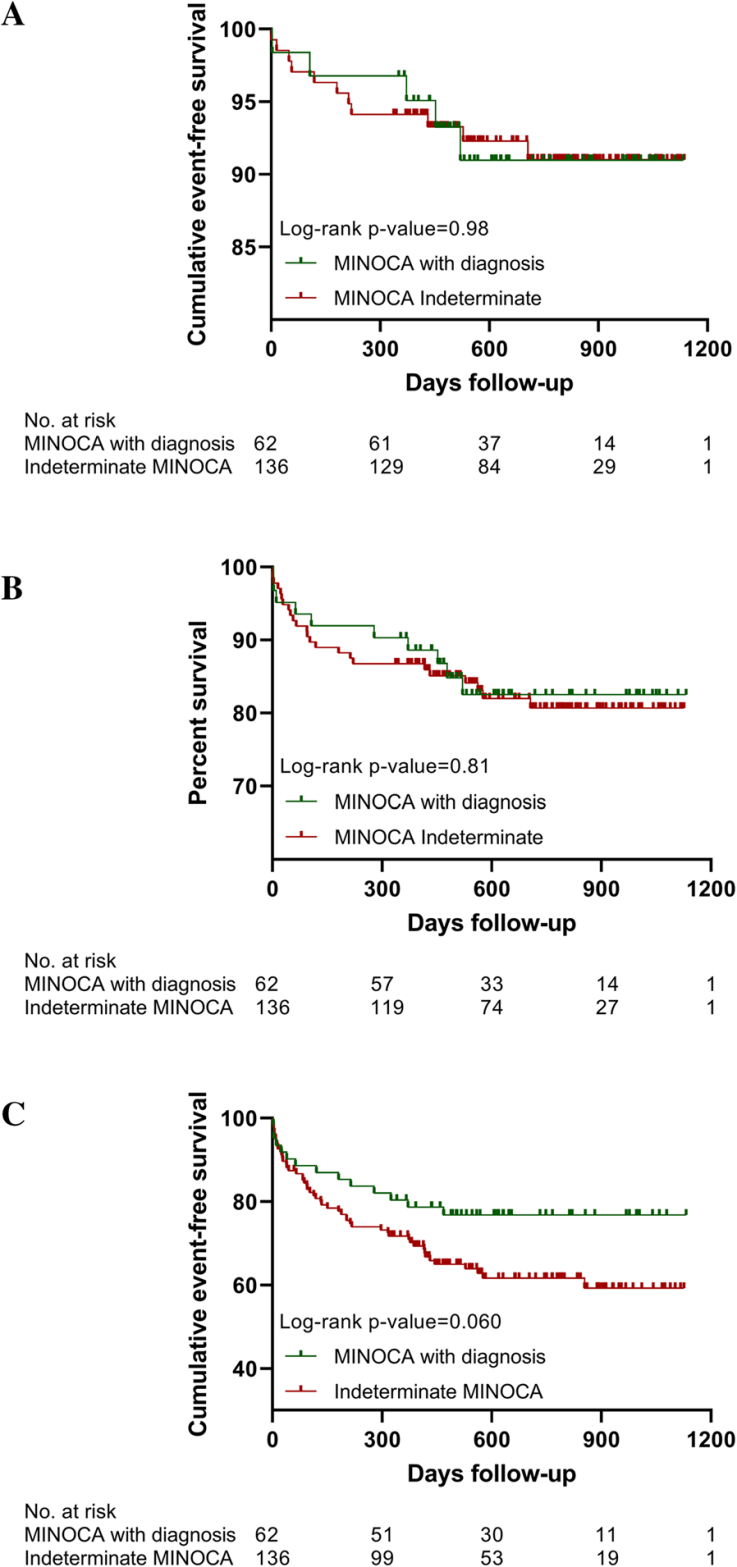
**a** Kaplan–Meier curves showing the cumulative event-free survival of MACE. **b** Represents the cumulative event-free survival of MACE including recurrent cardiac hospitalisation. **c** Represents the cumulative event-free survival of recurrent visits at the cardiac emergency department

Survival analysis for the time to MACE occurrence (hazard ratio (HR) for the indeterminate MINOCA group, 0.99; 95% CI 0.34–2.85; *p* = 0.98) and MACE including unplanned cardiac hospitalisation (HR for the indeterminate MINOCA group, 1.09; 95% CI 0.52–2.26; *p* = 0.81) showed no statistical differences between both MINOCA groups (Fig. 2a, b).

Furthermore, recurrent CED visits were more common in the indeterminate MINOCA group (36.8% vs 22.6%, *p* = 0.048) with a median time until the first recurrent visit of 118 days [25–321]. In the total cohort, only one MACE and one CED representation occurred earlier prior to concluding a final or indeterminate diagnosis. In the MINOCA with diagnosis group, half of the recurrent CED visits occurred in patients with confirmed AMI. In 81.3% of MINOCA patients, a cardiac origin could be identified: in 10.9% a non ST-elevation myocardial infarction (non-STEMI) was found, in 3.1% unstable angina pectoris, in 35.9% stable angina pectoris, in 14.1% a cardiac arrhythmia, and in 10.9% a heart failure event, while 6.3% showed a miscellaneous cardiac cause for the presentation. Reasons for recurrent CED visits did not differ between MINOCA groups.

In addition, patients from the indeterminate MINOCA group were significantly less frequently referred to cardiac rehabilitation (26.5% versus 43.5%, *p* = 0.017).

### Predictive factors of finding an underlying cause of MINOCA

Regression analysis was used to identify factors contributing to the identification of an underlying cause of the MINOCA event as well as recurrent CED visits.

The following factors were factors significant were associated with finding the underlying cause of the MINOCA event in univariable analysis: younger age, the absence of previous AMI, previous PCI, higher troponin-T and CK levels, ST-segment deviation, reduced LVEF and regional wall motion abnormalities, absence of coronary atherosclerosis, and performance of additional diagnostic tests including echocardiography; CMR, CT-angiography and left ventriculography.

Each variable depicted in the Table 3 was analysed in a separate multivariable logistic regression model with the following covariates: age, gender, hypertension, hypercholesterolemia, previous myocardial infarction, previous percutaneous coronary intervention, gout/rheumatic arthritis. This showed that higher levels of troponin-T and CK, ST-segment deviation, reduced LVEF, regional wall motion abnormalities, and employment of additional diagnostic measures (CMR, CT-angiography and left ventriculography) were independent predictors in finding the underlying MINOCA mechanism.

**Table 3:** Multivariable regression analysis for predictors of finding an underlying cause for the MINOCA event

	Adjusted OR^a^	95% CI	*p* value
hsTnT, per 100 ng/L increase	1.18	1.06–1.31	0.003
CK, per 100U/L increase	1.11	1.01–1.23	0.036
ST-deviation at presentation	2.92	1.47–5.78	0.002
Absence of coronary atherosclerosis on ICA	1.79	0.84–3.85	0.13
Echocardiography performed	2.03	0.86–4.77	0.11
LVEF ≤ 45%	7.95	2.77–22.8	< 0.001
Wall motion abnormalities	7.05	2.89–17.23	< 0.001
Additional imaging (composite of CMR, CT-angiography, left ventriculography)	9.18	4.38–19.26	< 0.001
CMR performed	6.53	2.87–14.89	< 0.001
CT-angiography performed	4.05	1.55–10.54	0.004
Left ventriculography performed	2.90	1.11–7.54	0.029

hsTnT: high-sensitivity troponin T; CK: creatine kinase; ICA: invasive coronary angiography; LVEF: left ventricular ejection fraction; CMR: cardiac magnetic resonance imaging; CT-angiography: computed tomography angiography

^a^Covariates used for correction: age, gender, hypertension, hypercholesterolemia, previous myocardial infarction, previous percutaneous coronary intervention, gout/rheumatic arthritis

### Predictive factors of recurrent Cardiac Emergency Department visits

Univariable analysis showed that a history of atrial fibrillation and treatment with oral anticoagulation resulted in significant more recurrent CED visits, whereas a conclusive MINOCA diagnosis, performance of solely CMR or the combination of CMR, CT-angiography and left ventriculography, resulted in fewer recurrent CED visits.

Each variable depicted in the Table 4 was analysed in a separate multivariable logistic regression model with the following covariates: age, gender, BMI, current smoker, previous myocardial infarction, atrial fibrillation. The performance of additional imaging (composite of CMR, CT-angiography, left ventriculography) and the prescription of ACE-inhibitors/ARBs remained significant predictors of fewer recurrent CED events. In patients with a conclusive MINOCA diagnosis, a trend to fewer recurrent CED visits was observed (OR 0.53; 95% CI 0.25–1.10; *p* = 0.088). The same trend was observed in survival analysis as presented in Fig. 2c (HR for the indeterminate MINOCA group as compared to the MINOCA with diagnosis group, 1.75; 95% CI 1.04–2.95; *p* = 0.060).

**Table 4:** Multivariable regression analysis for predictors of recurrent visits at the Cardiac Emergency Department

	Adjusted OR^a^	95% CI	*p* value
hsTnT, per 100 ng/L increase	0.99	0.94–1.04	0.63
CK, per 100U/L increase	1.01	0.93–1.09	0.86
ST-deviation at presentation	0.61	0.32–1.14	0.12
Absence of coronary atherosclerosis on ICA	0.78	0.39–1.55	0.48
Echocardiography performed	0.76	0.36–1.58	0.46
LVEF ≤ 45%	1.50	0.56–4.02	0.42
Wall motion abnormalities	0.74	0.32–1.76	0.50
Additional imaging (composite of CMR, CT-angiography, left ventriculography)	0.42	0.21–0.85	0.016
CMR performed	0.43	0.17–1.09	0.076
CT-angiography performed	0.60	0.22–1.63	0.31
Left ventriculography performed	0.38	0.12–1.20	0.097
MINOCA with diagnosis	0.53	0.25–1.10	0.088
*Medication at discharge*			
DAPT	1.52	0.78–2.93	0.22
Oral anticoagulation	1.57	0.47–5.27	0.47
Beta-blocker	0.85	0.44–1.63	0.62
ACE-inhibitor or ARB	0.51	0.27–0.97	0.039
Statin	0.97	0.48–1.96	0.93

hsTnT: high-sensitivity troponin T; CK: creatine kinase; LVEF: left ventricular ejection fraction; CMR: cardiac magnetic resonance imaging; CT-angiography: computed tomography angiography; MINOCA: myocardial infarction with non-obstructive coronary arteries; DAPT: dual antiplatelet therapy; ACE: angiotensin-converting enzyme; ARB: angiotensin II receptor blocker

^a^Covariates used for correction: age, gender, BMI, current smoker, previous myocardial infarction, atrial fibrillation

### Discharge medication

The risk of a recurrent CED visit (HR, 0.60; 95% CI 0.37–0.98; *p* = 0.038), occurrence of MACE (HR, 0.25; 95% CI 0.080–0.77; *p* = 0.009), and occurrence of MACE including unplanned cardiac hospitalisation (HR, 0.40; 95% CI 0.20–0.81; *p* = 0.008) was significantly lower in MINOCA treated with ACE-inhibitors/ARBs. Besides, in patients treated with DAPT, a significant reduction of MACE including unplanned cardiac hospitalisation was observed (HR, 0.44; 95% CI 0.22–0.88; *p* = 0.017), which was driven by the prescription of P2Y12-inhibitors (HR, 0.45; 95% CI 0.23–0.89; *p* = 0.021). Other prescribed medications were not associated with differences in endpoints.

## Discussion

In this study, we found that merely one-third of MINOCA patients an underlying cause for the event could be identified. Significantly more recurrent CED visits were observed in MINOCA without a conclusive diagnosis, while the occurrence of cardiovascular events was similar in both groups.

The exact prevalence of MINOCA varies across studies, ranging from 1 to 14%[11]. In the present study, the prevalence of the working diagnosis MINOCA was 6.6% of the total ACS population, which is in line with other recent studies [11, 14]. The underlying cause for the acute presentation could be identified in only one-third of the MINOCA population. The most prevalent underlying cause was AMI (38.7%), followed by cardiomyopathy (27.4%), (peri)myocarditis (22.6%), and miscellaneous (11.3%), showing that non-coronary causes for the MINOCA event were most prevalent, similar to previous research [3, 11, 15].

Most patients in our cohort (68.7%) were part of the indeterminate group, which is in line with Safdar et al. and Abdu et al. whom were unable to demonstrate a final diagnosis in 75% and 57% of MINOCA patients, respectively [8, 9]. Our observation of a relatively small proportion of patients with a conclusive diagnosis can be partially explained by the exclusion of patients stigmatized as having suffered from an AMI due to a coronary thromboembolism or coronary artery spasm without diagnostic confirmation by intravascular imaging or intracoronary provocation testing. When implementing those invasive techniques alone, the presence of plaque disruption/intracoronary thrombus or coronary artery spasm is expected to be detected in almost half of patients [4, 5, 16].

Although the current study showed that employment of additional diagnostic measures is related to a higher chance of identifying the underlying cause of the acute event, current clinical practice is not yet adjusted to this finding; CMR was performed in merely 38 cases (19.2%). We found that in 50% of these patients, a conclusive diagnosis was found. Previous studies have shown a similar diagnostic yield of CMR in MINOCA, ranging from 50 to 74%[3, 6, 17–19]. Although CMR is recommended to be performed rather soon following clinical presentation, since myocarditis and Takotsubo syndrome tend to resolve in 2–4 weeks, the median time to CMR in our population was 4 weeks. This might have contributed to a lower than expected diagnostic accuracy and yield [20, 21].

Future management strategies in MINOCA should focus on a combined approach of revealing the underlying MINOCA mechanism with direct intravascular imaging and early CMR. Confirmation of the underlying diagnosis is crucial since MINOCA may not be considered benign [10]. Recently, Reynolds et al. demonstrated the value of combining a multi-modality imaging strategy with CMR and optical coherence tomography (OCT) which showed a definitive MINOCA mechanism in 84.5% of female MINOCA patients [16]. The position of intracoronary vasomotor and resistance testing in a diagnostic algorithm needs to be confirmed, and could be of added value during a second procedure in the remaining MINOCA patients.

During follow-up, MACE occurred in 17 patients (8.6%), while in previous studies the incidence of MACE ranged between 4.9 and 24%[9, 14, 22]. Different classifications of MINOCA, a small sample size and short follow-up times may explain this broad range. No significant difference in the occurrence of MACE between MINOCA patients with and without a conclusive diagnosis were found.

However, we found that 32% of MINOCA cases showed at least one recurrent CED visit with a median time-to-event of three months. Interestingly, those without a conclusive diagnosis showed significantly more frequent CED visits. This may reflect the presence of illness uncertainty that patients without a conclusive diagnosis may experience following hospital discharge. Besides, patients from this MINOCA subgroup were significantly less often referred to the cardiac rehabilitation programme, which may seem logical since the underlying cause for the acute presentation remained unknown, but may have contributed to recurrent complaints and concomitant CED visits.

Almost half of the patients with a recurrent CED visit presented with chest pain suggestive of a cardiac origin. Although persistent cardiac chest pain in patients without obstructive coronary artery disease has shown to be a predictor for adverse cardiac events, data on recurrent chest pain in MINOCA patients remains scarce [23], though Jedrychwoska et al. demonstrated that recurrent chest pain occurred in almost one-fifth MINOCA [24]. Moreover, 12.1% of our MINOCA population had a recurrent hospitalisation at the cardiology department. Similar results were observed by Abdu et al., reporting a cardiovascular-related rehospitalisation rate of 13.8% at 1-year follow-up in MINOCA, whereas Jedrychowska et al. reported 17.1% recurrent hospitalisations [9, 24]. Thus, although significant coronary obstructions are ruled out in the MINOCA population, preliminary data show that these patients are suffering from persistent complaints with risk of future hospitalizations.

In the present study, lower age, the absence of a previous AMI, ST-segment deviation, reduced LVEF, higher levels of CK and troponin-T, and use of additional imaging tools were predictors of finding an underlying cause for the MINOCA event. Interestingly, performing additional imaging was also related to less frequent recurrent CED visits. This underlines the importance and role of employment of additional imaging modalities in MINOCA patients, primarily to reveal the underlying diagnosis, secondarily to prevent recurrent CED presentations and perhaps recurrent hospitalisations.

Given the fact that MINOCA may show a range of possible underlying mechanisms, the current conventional secondary prevention strategy for AMI may not be suitable for all patients. We found that in the indeterminate MINOCA group, P2Y12-inhibitors were prescribed more often, which seems logical since in our confirmed MINOCA cohort this particular medication is not always necessary. In previous research, Lindahl et al. demonstrated the long-term beneficial effects of treatment with statins and ACE-inhibitors/ARBs, whereas a neutral effect of DAPT and statin therapy was found [25]. We observed fewer recurrent CED visits, MACE and MACE including unplanned cardiac hospitalisation in MINOCA patients treated with ACE-inhibitors/ARBs, whereas the prescription of DAPT only led to reduced MACE including unplanned cardiac hospitalisation, with no effect on other endpoints. However, these results should be interpreted with caution due to the relatively small sample size.

Several limitations should be considered. First, the generic definition of MINOCA used in this study includes patients with a working diagnosis of MINOCA at the time of coronary angiography, meaning that patients with non-coronary causes for the acute presentation were also included. Although this does reflect current clinical practice, the Fourth Universal Definition state the term MINOCA should be exclusively applied to those with an ischaemic mechanism responsible for the myocyte injury [12]. Since the indeterminate MINOCA group was expected to be heterogenic, we decided to combine all confirmed underlying causes in one “MINOCA with diagnosis” group, reflecting actual clinical practice, rather than include only patients with confirmed AMI. Moreover, this approach allows for comparison of the current study results with previous studies, as most studies describe the MINOCA population in a similar manner. Whilst interpreting the results, it is important to acknowledge the possibility that some of the indeterminate MINOCA group patients did receive a specific diagnosis after the observed follow-up time. With a follow-up of 719 [504–888] days, this is, however, relatively unlikely.

Secondly, albeit previous studies have shown the effectiveness and usefulness of CMR and additional intracoronary testing (i.e., intravascular ultrasound, optical coherence tomography or provocative spasm testing), only few patients underwent these extensive assessments. Besides, in 9 patients, CMR was performed > 2 months after the index presentation which could have had a negative impact on the chance of finding an underlying diagnosis. Although the awareness of MINOCA as a separate group of patients with suboptimal clinical outcomes continues to grow, a standard diagnostic work-up for MINOCA is still lacking.

Third, inherent to the retrospective nature of the study design, a relatively small sample size and data collection by record review, some degree of information bias or confounding might play a role.

Fourth, clinical event reporting could be underestimated since, due to privacy regulations, we were not allowed to contact the general practitioner or other nearby hospitals to improve event reporting.

## Conclusion

In merely one-third of MINOCA patients a conclusive diagnosis for the acute presentation could be identified. Recurrent CED visits were more often observed in indeterminate MINOCA patients, while the occurrence of cardiovascular events was similar in MINOCA patients with and without a definitive diagnosis.

## Data Availability

The dataset supporting the conclusions of this article is available upon request by contacting the corresponding author.

## Abbreviations

ACE: Angiotensin-converting enzyme
ACS: Acute coronary syndrome
AMI: Acute myocardial infarction
ARB: Angiotensin-II receptor blockers
CED: Cardiac Emergency Department
CK: Creatine kinase
CMR: Cardiac magnetic resonance imaging
CT-angiography: Computed tomography angiography
DAPT: Dual antiplatelet therapy
HR: Hazard ratio
ICA: Invasive coronary angiography
IQR: Interquartile range
LVEF: Left ventricular ejection fraction
MACE: Major adverse cardiac events
MINOCA: Myocardial infarction with non-obstructive coronary arteries
Non-STEMI: Non ST-elevation myocardial infarction
OR: Odds ratios
PCI: Percutaneous coronary intervention
SD: Standard deviation
